# Effects of a health-seeking behavior education program based on motivational interview techniques on health-seeking behaviors, illness self-management, and anxiety in elderly immigrant women: a randomized controlled study

**DOI:** 10.3389/fpsyg.2025.1547195

**Published:** 2025-03-18

**Authors:** Pınar Harmancı, Yasemin Sazak, Semra Bulbuloglu

**Affiliations:** ^1^Nursing Department, Faculty of Health Science, Kahramanmaraş Istiklal Üniversitesi, Kahramanmaraş, Türkiye; ^2^Division of Surgical Nursing, Nursing Department, Faculty of Health Sciences, Istanbul Aydin University, Istanbul, Türkiye

**Keywords:** migrant, elderly, woman, motivational interview, health-seeking behavior, anxiety

## Abstract

**Introduction:**

Whether voluntary or forced, migration always affects those who migrate. Migrants experience the negative effects of migration more severely if they are older, chronic patients, or women. If a group of migrants possess all these vulnerabilities, they need more assistance and healthcare.

**Objective:**

This study was carried out to investigate the effects of a health-seeking behavior education program based on motivational interview techniques on health-seeking behaviors, illness self-management, and anxiety in elderly migrant women.

**Material and method:**

This study was conducted with a randomized controlled experimental design involving an experimental group (*n* = 18) and a control group (*n* = 18). The simple random sampling method was chosen to ensure that the groups were randomly distributed and had the same number of participants. A “Personal Information Form,” the “Health-Seeking Behaviors Scale,” the “Health Anxiety Inventory,” and the “Chronic Illness Self-Management Scale” were used to collect data. The intervention consisted of four structured seasons. In addition, the main themes were also covered with Motivational Interviewing (MI) techniques within these seasons.

**Results:**

Before the intervention (pretest), no significant differences were found between the groups in health-seeking behaviors, health anxiety, or chronic illness self-management (*p* > 0.05). In the posttest, the control group showed a significant decrease in total Health-Seeking Behaviors Scale scores and in professional and traditional health-seeking behaviors (*p* < 0.05), while online health-seeking behaviors did not significantly change (*p* > 0.05). Additionally, the control group's self-stigma and other chronic illness self-management dimensions worsened (*p* < 0.05). In contrast, the experimental group showed significant improvements in total health-seeking behaviors, particularly in online and professional behaviors (*p* < 0.05). They also showed reductions in health anxiety and self-stigma, with improvements in chronic illness self-management dimensions such as coping with stigma and treatment adherence (*p* < 0.05). Intergroup comparisons revealed that the experimental group had significantly better outcomes in all these areas compared to the control group (*p* < 0.05).

**Discussion and conclusion:**

The study highlights that migration negatively affects health-seeking behaviors, especially in elderly migrant women. However, the health-seeking behavior education program based on motivational interviewing techniques proved to be effective in improving these behaviors, illness self-management, and reducing anxiety. This suggests the need for targeted interventions to support vulnerable migrant populations.

## Introduction

Migration is defined as the movement of a person or a group of people within a state or across international borders. In other words, migration involves population movements that occur as a result of the mobility of people without regard to its cause, duration, or structure. The movement processes of individuals who migrate for various reasons such as those who are forced to leave a place, refugees, and economic migrants are also defined as migration. Individuals who migrate are called migrants (Perruchoud and Redpath-Cross, 2013). Migration may be voluntary or forced. In situations such as natural disasters and wars, individuals may be forced to migrate. This situation may lead individuals to migrate unprepared, become exposed to health problems, and experience difficulties in accessing health services at their destination. Thus, migrants constitute a disadvantaged group that needs to be supported in terms of their health-related needs. Turkey is among the countries in the world hosting the highest numbers of migrants. In Turkey, there are about 5 million migrants including ~3.7 million Syrians who have been given “temporary protection” status (Ertem and Keklik, 2019). According to the 2022 International Migration Report of TURKSTAT (Turkish Statistical Institute), within the population of people who arrived in Turkey through migration, 48.1% of those arriving in 2021 and 47.1% of those arriving in 2022 were women (TÜIK, 2022).

Health-seeking behaviors refer to the health-related activities and behaviors of individuals who do not feel well, have disease symptoms, or are seeking medical assistance. Health-seeking behaviors affect the treatment, diagnosis, and recovery processes of individuals. Individuals may be in search of a way to alleviate or resolve their symptoms. This search may result in behaviors such as visiting healthcare institutions, following the recommendations of people that one trusts, looking for information on the internet, and self-medication (Deniz and Çimen, 2021; Huang et al., 2019). In the literature, it has been stated that in comparison to the local population, individuals who migrate have significantly higher rates of visiting emergency services and significantly different behaviors in terms of issues such as seeking medical assistance (Breton et al., 2017; Krzyż and Lin, 2024), and they have a poorer state of health in general (Moullan and Jusot, 2014). Regardless of its reason, migration influences individuals psychologically, socially, and physically. Factors such as situations leading to migration, the attitudes of the general public toward migrants, problems experienced in access to services, health-related beliefs, and the conditions of the destination region may affect the adjustment process of individuals. In particular, among disadvantaged groups, the elderly, the disabled, women, and children constitute the risk groups that are affected by the migration and adjustment process the most (Arabaci et al., 2016). For this reason, elderly migrants are more susceptible to health problems due to frailty, physiological disorders, their weakened immune system, chronic diseases, and chronic psychiatric illnesses. This leads to an increase in their health-related needs and a decrease in their capacity to self-manage illness. Therefore, the inability of elderly migrant women to access healthcare services to a sufficient extent results in the development of psychological issues, physical problems, and mental problems like anxiety and depression, as well as an increase in the prevalence of mortality in this population (Breton et al., 2017). In elderly migrant women, in particular, significant mental problems are brought about by leaving the land they were born and grew up due to various challenges, difficulties in meeting a different culture and a different language toward the end of their lives, the absence of familiar daily routines, and leaving their familiar social circumstances. Chronic physical and mental illnesses may also develop faster in such a vulnerable group (Derose et al., 2007; Guruge et al., 2015; Guruge and Matsuoka, 2022).

Motivational interviewing is a patient-centered approach focusing on behavioral change that had initially been developed in the field of addiction but is also used in the improvement and promotion of health (Morton et al., 2015). Previous studies have revealed that education programs based on motivational interviews improve the self-management of illness, develop positive health-related behaviors (Morton et al., 2015), and reduce depression and anxiety (Li et al., 2020; Zahra et al., 2024). Elderly migrant women may experience deficiencies in coping with illnesses due to the effects of migration and their health conditions. They also constitute a disadvantaged group in the psychological sense. This affects their help-seeking behaviors and prevents them from developing these behaviors effectively. This study was carried out to investigate the effects of a health-seeking behavior education program based on motivational interview techniques on health-seeking behaviors, illness self-management, and anxiety in elderly migrant women with chronic diseases.

### Hypotheses

**H1**_**1**_**:** The health-seeking education program carried out with elderly migrant women with chronic diseases based on motivational interview techniques will improve their health-seeking behaviors.
**H2**_**1**_**:** The health-seeking education program carried out with elderly migrant women with chronic diseases based on motivational interview techniques will increase their capacity to self-manage illness.
**H3**_**1**_**:** The health-seeking education program carried out with elderly migrant women with chronic diseases based on motivational interview techniques will reduce their anxiety levels.

## Materials and methods

### Design and sampling

This study was carried out with a randomized controlled experimental design (ClinicalTrials.gov, NCT06505707). The population of the study included elderly migrant women aged 65 or older who had chronic diseases and were registered at a Provincial Directorate of Migration in the Mediterranean Region. The study was conducted between 8 July 2024 and 16 August 2024. Because the population was small, it was aimed to include all individuals who met the inclusion criteria in the sample. The sample of the study consisted of 36 participants (control group: 18, experimental group: 18).

**Figure 1:**
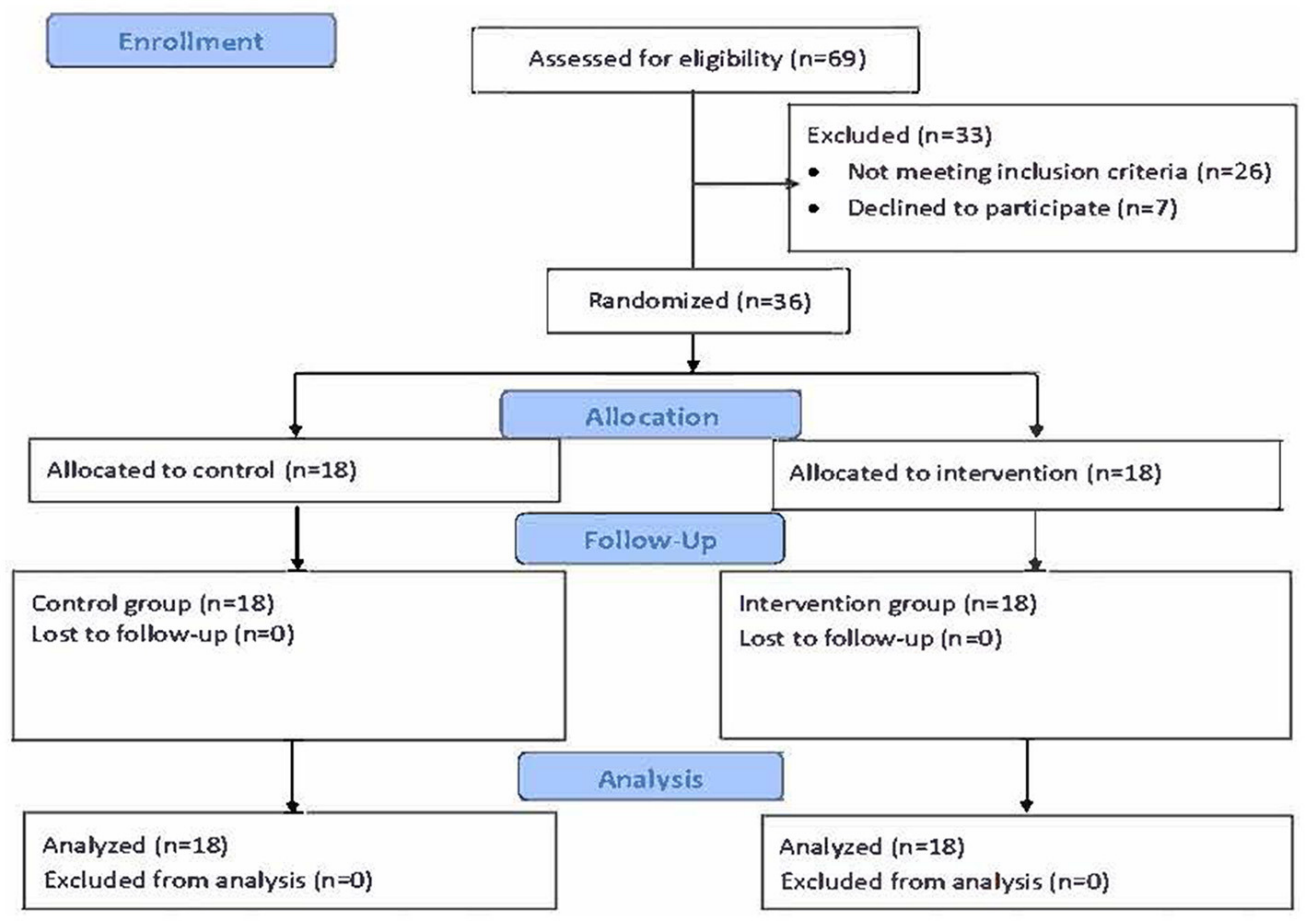
Consort flow diagram.

The simple random sampling method was chosen to ensure that the groups were randomly distributed and had the same number of participants. Participants meeting the inclusion criteria were numbered and listed. A randomization list was then created on a website (https://www.randomizer.org/, Date Accessed: 27.06.2024). The participants with the first numbers that were randomly selected constituted the experimental group, while the remaining participants were allocated to the control group. The random allocation of the participants to the groups was performed by the researchers. The CONSORT flow diagram of the study regarding the inclusion of the participants is shown in Figure 1.

The inclusion criteria were being a migrant woman who is 65 years old or older, having a chronic disease, having lived in Turkey for at least 6 months without prior Turkish citizenship, having or not having gained Turkish citizenship later, not having a communication problem, and agreeing to participate in the study. The sample of the study excluded women who had dementia, Alzheimer's, or a diagnosed psychiatric illness and those who had Mini-Mental Examination scores below 24. The participants who were absent in at least two motivational interviews and those who could not be contacted for the posttest were removed from the sample.

### Power analysis

Because the population was small, it was aimed to reach the entire population. Participants were included in the sample based on the inclusion criteria without using a sampling method. For this reason, the power of the study was calculated by a *post-hoc* power analysis using the G^*^Power 3.1.9.4 program (Faul et al., 2009). The power of the study based on the total Health-Seeking Behaviors Scale scores of the participants (Table 4) was found to be 1.0.

### Data collection instruments

#### Personal information form

The form, which was developed by the researchers in light of the relevant literature, included questions about the sociodemographic and disease-related characteristics of the participants.

#### Health-Seeking Behaviors Scale

HSBS was developed by Kiraç and Öztürk (2021) to evaluate the health-seeking behaviors of individuals. It consists of 12 items and three dimensions named online health-seeking behaviors, professional health-seeking behaviors, and traditional health-seeking behaviors. The total score of the scale is obtained by dividing the sum of scores of all items (range: 12–60) by the number of items (12), and it ranges from 1 to 5. Higher scores indicate higher levels of health-seeking behaviors. Cronbach's alpha coefficients were reported as 0.726 for “online health-seeking behaviors,” 0.720 for “professional health-seeking behaviors,” 0.736 for “traditional health-seeking behaviors,” and 0.755 for the overall scale (Kiraç and Öztürk, 2021). In this study, Cronbach's alpha coefficients for the scale were found to be 0.838 in the pretest and 0.926 in the posttest.

#### Health Anxiety Inventory

HAI is a self-report scale consisting of 18 items which was developed by Salkovskis et al. (2002). The Turkish validity and reliability study of the scale was carried out by Aydemir et al. (2013). In the validity and reliability study of the scale, its Cronbach's alpha coefficient was reported as 0.918. Fourteen items of the scale are items with four options each, questioning the mental state of the respondent. The four remaining items ask the respondent to assume that they have a serious illness and think about how their mental state would be in such a situation. Each item has a score range of 0–3, and the total score of the scale varies between 0 and 54. Higher scores indicate higher levels of health anxiety (Aydemir et al., 2013). In this study, Cronbach's alpha coefficients for the scale were found to be 0.902 in the pretest and 0.997 in the posttest.

#### Chronic Illness Self-Management Scale

CISM, which was developed by Ngai et al. (2020), was tested for validity and reliability in Turkish by Öztürk et al. (2021). The Turkish version of the scale consists of 23 items and 4 dimensions. The dimensions of the scale are self-stigma, coping with stigma, health maintenance efficacy, and treatment adherence. It is a 5-point Likert-type scale, where each item is scored from 1 to 5. The items in the treatment adherence dimension of the scale are inversely scored. Cronbach's alpha coefficients were reported as 0.876 for self-stigma, 0.850 for treatment adherence, 0.820 for coping with stigma, and 0.789 for health maintenance efficacy. The total score of the scale is calculated between 1 and 5 (Öztürk et al., 2021). In this study, Cronbach's alpha coefficients were calculated as 0.967 and 0.997 for self-stigma, 0.796 and 0.979 for treatment adherence, 0.958 and 0.995 for coping with stigma, and 0.954 and 0.987 for health maintenance efficacy in the pretest and posttest, respectively.

#### Implementation

The researcher who would perform the motivational interview (MI) interventions participated in “Motivational Interview Training” and received a certificate.

The participants were informed about the Health-Seeking Behavior Education Program Based on Motivational Interview Techniques. After the allocation of the participants to the experimental group (*n* = 18) and the control group (*n* = 18) by simple random allocation, the pretest forms (Personal Information Form, HSBS, HAI, CISM) were administered to both groups via the face-to-face interview method, and the days on which the Health-Seeking Behavior Education Program Based on Motivational Interview Techniques would be carried out with the participants in the experimental group were determined. The participants in the experimental group were divided into 4 groups for the MI sessions, in which the researcher met the individuals in each of the 4 groups individually for 4 days a week. The MI sessions were held face-to-face 4 days a week (Monday, Tuesday, Thursday, and Friday) for 4 weeks, and each MI session in the Health-Seeking Behavior Education Program Based on Motivational Interview Techniques lasted ~30–35 min. The participants in the experimental group who were divided into 4 groups to receive education were called by the researcher and invited to a room at the university in which a therapeutic setting was provided. Interviews continued under these therapeutic conditions. For the intervention, there were 4 participants in Group 1, 4 in Group 2, 5 in Group 3, and 5 in Group 4. The researcher held interviews with each group on the days and at the hours that were arranged beforehand so that the face-to-face intervention was implemented in each group on 1 of the 4 days allocated in the week. After the completion of the Health-Seeking Behavior Education Program Based on Motivational Interview Techniques, the posttest forms (Personal Information Form, HSBS, HAI, CISM) were administered to both groups via the face-to-face interview method.

In the control group, posttest data were collected face-to-face by meeting the participants on a previously arranged day. Following the end of the data collection process, the participants in the control group were provided with the Health-Seeking Behavior Education Program Based on Motivational Interview Techniques in one session (Table 1). No adverse effects were observed in the study.

**Table 1:** Health-seeking behavior education program based on motivational interview techniques.

**Session subjects and content**		
**Session**	**Subject**	**Motivational interview technique and objective**
**Session 1**	•The participants are given information about obtaining health-related information on the internet and its risks. •The following questions are directed at the participants in the given order: °What could be the health problems that may be caused by health information obtained from the internet? ° What could be the health problems that may be caused by television programs and health warnings? ° What could be the health problems that may be caused by health recommendations found on social media? •The researcher emphasizes the importance of accessing healthcare institutions and asking for assistance from a healthcare service provider in cases of illness and curiosity about a health-related behavior. •The researcher explains how the education to be provided to the participants about internet searches and television programs about health will affect their awareness regarding their disease, their medication usage, and the health behaviors they will keep in mind while managing their disease (e.g., sleep, exercise, nutrition). The participants are informed that health recommendations encountered on social media are based on personal experiences (if there is a social media recommendation strongly embraced by the participant, it is addressed using motivational interview techniques).	•Introduction •Open-ended questions about the general characteristics of the participants are asked to know about them. •Open-ended questions about the usage of the internet and social media by the participants are asked. •The information obtained about the participant is reviewed, and it is ensured that she also reassesses this information (reflective listening). •The perception of health-disease is evaluated, open-ended questions are asked to allow the participant to review this perception herself, and effective listening and reflective listening methods are used. •Open-ended questions are asked about the usage of the internet, television programs, and social media by the participants to solve their health problems. •The effects of their usage of the internet, television programs, and social media on the disease management and anxiety levels of the participants are evaluated on a scale of 1–10, and to create awareness, it is ensured that the participants talk about the consequences of their negative behaviors.
**Session 2**	•The participants are asked whether they visit family health centers. •The participants are informed about the health problems for which they can visit family health centers and those that can be prevented by visiting these centers. •The effects of family medicine practices on health outcomes in the elderly population are discussed.	•Information about the participant in the context of the topics of the session is obtained by asking open-ended questions. Active listening is used. •The experiences of the participants are summarized (which will allow the participants to perform an overview and potentially constitute an important technique for the transformation of things that they may want to change).
	•Using examples, the researcher explains how family medicine practices can affect the myths in society regarding the health of women, the elderly, and migrants. •The researcher finds out if there are participants who have visited family health centers before but have not adhered to the recommendations of family physicians and explains the importance of these recommendations. •The participants are given information regarding health-related topics about which they can request education from nurses working at family health centers, and they are encouraged to seek education. •The participants are given information regarding the types of counseling they can receive from nurses working at family health centers about daily healthy lifestyle behaviors.	•In this session, the reflective listening and summarization techniques will offer an important opportunity to uncover “dilemmas.” •If dilemmas about visiting family health centers and receiving counseling from a physician or nurse are revealed (including anxiety), the participants are ensured to notice these dilemmas using the 1–10 scale technique. •In this session, visiting family health centers for their “current health problems,” if any, will be given as an assignment to the participants. The participants will be asked to receive counseling by asking the family physician and nurse questions regarding the health behaviors about which they want to learn.
**Session 3**	•The researcher evaluates whether the participants follow the treatment recommendations of their loved ones. •The participants are given information about the health risks of performing/not performing recommendations of loved ones about healthy lifestyle behaviors. •The participants are asked whether they have tried herbal medicines and whether they have a plant-based practice they currently maintain. •Myths about health behaviors specific to their culture are discussed. •The dilemmas of the participants about these issues are identified and discussed (motivational interview techniques are used in a way suitable for group practice).	•The dilemmas identified in the previous week are discussed at this stage. •The direction of the change experienced following the 1st home assignment of the participant is evaluated objectively (open-ended questions are asked, and summarization is made). •Information about the topics assigned to the session is outlined via open-ended questions. •If there are any culture-specific myths, they are identified by effective listening and reflective listening. The 1–10 scale technique is used for dilemmas. Using this technique is important for creating awareness regarding these dilemmas. •Any persistent thoughts remaining from the previous weeks, if any, are identified, open-ended questions are asked, and the summarization technique is used. •The participants are reminded of the health problems about which they can receive more appropriate assistance when they practice “positive health behaviors.”
		•The effects of positive health behaviors on disease management are discussed, and effective listening is performed. Awareness is raised by summarization (this will allow the participants to review their situation).
**Session 4**	•The researcher asks whether the participants have received recommendations from those “who also experienced the same disease” before. •The recommendations they have received from those who also experienced the same disease and their opinions about these recommendations are evaluated. Myths, if any, are discussed, and motivational interview techniques are used to resolve dilemmas.	•As topics for the session of the week, current problems are identified by asking open-ended questions. •The topics in which there has been a change are explained. •The disease self-management statuses and anxieties of the participants are addressed again. •The difference between myths and facts is clearly discussed, and the summarization method is used. •Changes in dilemmas are discussed, and agreements are made if necessary.

Intervention content session 1; Information was given about the use of the internet and its risks in obtaining health information; open-ended questions and active listening were used as motivational interviewing techniques in this step. This stage constituted an important season for both training and information gathering. In session 2, the sample was asked to ” They were asked whether they use “Community Health Centers” in Turkey and information was provided. At this stage, ambivalences were revealed, open-ended questions were asked and summarized, and a homework assignment was given (they were asked to go to the Community Health Center for any health problems, to ask for information, and to follow up their treatment, if any). In session 3, it was evaluated whether the sample took into account the treatment recommendations, whether they complied with the treatment, and information was given for treatment compliance. At this stage, the homework given in the previous week was evaluated. By using techniques such as open-ended questions and summarizing, “changing thoughts and feelings” were discussed. In the last session, the sample's experiences with “others who had the same experiences” were learned. In this season, socratic questioning and the subjects of change were discussed, and myths-truths and permanent changes were discussed.

#### Data analysis

The IBM SPSS 25 package program was used to analyze the collected data. Descriptive statistics including frequency, percentage, mean, and standard deviation values were calculated. The Kolmogorov-Smirnov test was conducted to test the normality of the distribution of the data. The categorical data of the groups were compared using Fisher's exact chi-squared tests in 2 × 2 and R × C tables (Mehta and Patel, 1986). Because the pretest and posttest scale scores of the groups were non-normally distributed, intergroup comparisons were made using the Mann-Whitney U test, while the intragroup comparisons of the pretest and posttest scores of each group were carried out using the Wilcoxon signed-rank test. Effect sizes were calculated based on effect size indices for non-parametric tests (r = Z/√N). Effect size values of 0.10, 0.30, and 0.50 were considered small, medium, and large effect sizes, respectively (Cohen, 1988). The threshold for statistical significance in the analyses was accepted as *p* < 0.05.

#### Ethical aspects of the study

Approval to conduct the study was obtained from the non-invasive studies ethics committee of a university (decision date: 24.04.2024, no: 2024/08-01). The participants were informed about the procedures of the study, and the written consent of those who agreed to participate in the study was received. Permissions were received to use the scales that were utilized to collect data in the study. The principles of the Declaration of Helsinki and publication ethics were followed throughout the study.

#### Limitations

As the study included a sample of women who had migrated to Turkey from certain regions, the results of the study may not be generalizable to all elderly migrant women with chronic diseases. It was not possible to reach a comprehensive sample size because of the specificity of the inclusion criteria.

## Results

It was determined that the women who participated in the study were homogeneously distributed between the experimental and control groups in terms of their age, BMI, marital status, education status, health insurance status, occupation, socioeconomic status, smoking status, regular medication usage status, health follow-up frequency, self-assessment of health, the extent to which their illness affected their lives, the number of chronic diseases they had, whether they monitored the symptoms of their diseases, the origin country from which they migrated to Turkey, and their reasons for migrating. On the other hand, the groups were non-homogeneously distributed in terms of the family types and family support statuses of the participants (Table 2).

**Table 2:** Descriptive characteristics (*N* = 36).

**Variables**	**Control (*n* = 18)**	**Experiment (*n* = 18)**	***Z***	***p***
	**X̄** ±**SD**	**X̄** ±**SD**		
Age (min.-max.)	74.78 ± 6.63 (65–84)	73.83 ± 6.91 (66–88)	−0.523	0.601
BMI (min.-max.)	29.78 ± 2.90 (25.0–38.95)	30.37 ± 6.97 (20.32–40.48)	−0.285	0.776
**Variables**	***n*** **(%)**	***n*** **(%)**	***p** ^†^*
**Marital status**			0.075
Married	3 (16.7)	9 (50)	
Widowed/divorced	15 (83.3)	9 (50)	
**Education**			0.229
Illiterate	18 (100)	15 (83.3)	
Literate (no formal degree)	0 (0)	2 (11.1)	
Primary-secondary school	0 (0)	1 (5.6)	
**Family type**			**0.029**
Nuclear family	11 (61.1)	6 (33.3)	
Extended family	1 (5.6)	8 (44.4)	
Living alone	6 (33.3)	4 (22.2)	
**Has health insurance**			0.486
Yes	18 (100)	16 (88.9)	
No	0 (0)	2 (11.1)	
**Occupation**			-
Not working/homemaker	18 (100)	18 (100)	
**Socioeconomic status**			-
Income less than expenses	18 (100)	18 (100)	
**Smoking status**			
Current smoker	0 (0)	1 (5.6)	0.658
Quitted smoking	4 (22.2)	2 (11.1)	
Never smoked	14 (77.8)	15 (83.3)	
**Uses medication regularly**			
Yes	7 (41.2)	8 (44.4)	1.000
No	10 (58.8)	10 (55.6)	
**Frequency of health follow-ups**		
Every 0–3 months	0 (0)	1 (5.6)	0.098
Once a year	0 (0)	1 (5.6)	
When she has a complaint	17 (94.4)	13 (72.2)	
Has not attended for a long time	1 (5.6)	3 (16.7)	
**Receives support from family**			**0.002**
Yes	2 (11.1)	4 (22.2)	
No	15 (83.3)	5 (27.8)	
Sometimes	1 (5.6)	9 (50.0)	
**Thinks her health is**			0.658
Moderate	0 (0)	1 (5.6)	
Poor	2 (11.1)	3 (16.7)	
Very poor	16 (88.9)	14 (77.8)	
**Her disease affects her life**			0.603
Moderately	1 (5.6)	3 (16.7)	
Severely	17 (94.4)	15 (83.3)	
**Number of chronic diseases**			0.688
One	5 (27.8)	4 (22.2)	
Two	9 (50.0)	7 (38.9)	
Three	4 (22.2)	7 (38.9)	
**Monitors her disease symptoms**			1.000
Yes	8 (44.4)	7 (38.9)	
No	10 (55.6)	11 (61.1)	
**Country of origin**		
Afghanistan	0 (0)	1 (5.6)	0.794
Palestine	2 (11.1)	3 (16.7)	
Iraq	1 (5.6)	0 (0)	
Syria	14 (77.8)	12 (66.7)	
Turkmenistan	1 (5.6)	2 (11.1)	
**Reason for migration**		
War	8 (44.4)	9 (50)	1.000
Socioeconomic reasons	2 (11.1)	2 (11.1)	
War and socioeconomic reasons	8 (44.4)	7 (38.9)	

Z, Mann-Whitney U test.

X̄, Mean; SD, Standard deviation.

^†^Chi-squared Fisher's exact test or Fisher-Freeman-Halton exact test.

*p* < 0.05 are in bold.

In the intergroup comparisons before the intervention (pretest), no statistically significant differences were found between the experimental and control groups in terms of their HSBS total and subscale scores, HAI total and subscale scores, or CISM total and subscale scores (*p* > 0.05, Table 3).

**Table 3:** Intergroup comparisons of pretest scores (*N* = 36).

	**Control (*****n*** = **18)**		**Experiment (*****n*** = **18)**		**Z**	***p***	**Effect size (r)**
	**X̄** ±**SD**	**Median (IQR)**	**X̄** ±**SD**	**Median (IQR)**			
**Health-Seeking Behaviors Scale**							
Total HSBS	1.57 ± 0.47	1.33 (0.71)	1.94 ± 0.60	2.04 (1.06)	−1.622	0.105	0.27
Online health-seeking behaviors	1.02 ± 0.08	1.00 (0.0)	1.11 ± 0.32	1.00 (0.0)	−0.660	0.509	0.11
Professional health-seeking behaviors	2.00 ± 0.90	1.67 (0.67)	2.54 ± 1.10	3.00 (1.75)	−1.383	0.167	0.23
Traditional health-seeking behaviors	2.26 ± 1.14	1.67 (1.50)	2.98 ± 1.40	3.17 (2.25)	−1.646	0.100	0.27
**Health anxiety inventory**							
Total HAI	46.22 ± 9.64	50.00 (6.75)	45.67 ± 5.68	44.50 (7.50)	−1.270	0.204	0.21
Excessive concern and anxiety about physical symptoms	35.11 ± 9.63	38.50 (8.75)	34.50 ± 5.61	34.00 (8.25)	−1.176	0.240	0.19
Negative consequences	11.11 ± 1.02	11.00 (1.25)	11.17 ± 1.42	11.50 (1.00)	−0.550	0.582	0.09
**Chronic illness self-management scale**							
Self-stigma	4.57 ± 0.90	5.00 (0.71)	4.53 ± 0.64	5.00 (1.0)	−0.417	0.676	0.06
Coping with stigma	1.60 ± 0.79	1.20 (0.90)	1.73 ± 0.49	1.70 (0.65)	−1.473	0.141	0.24
Health maintenance efficacy	1.40 ± 0.75	1.00 (0.50)	1.46 ± 0.58	1.00 (1.0)	−0.559	0.576	0.09
Treatment adherence	2.18 ± 0.97	2.00 (0.85)	2.22 ± 0.62	2.10 (0.65)	−0.856	0.392	0.14

Z, Mann-Whitney U test; X̄, Mean; SD, Standard Deviation.

IQR, Interquartile Range.

In the posttest, the control group had significantly lower total Health-Seeking Behaviors Scale scores and scores in the Health-Seeking Behaviors scale dimensions of professional health-seeking behaviors and traditional health-seeking behaviors compared to the pretest, and this difference had a large effect size (*p* < 0.05). On the other hand, the online health-seeking behaviors dimension scores of the control group did not significantly differ between the pretest and the posttest (*p* > 0.05). The scores of the control group in the self-stigma dimension of the Chronic Illness Self-Management Scale were significantly higher in the posttest compared to the pretest, and this difference had a large effect size (*p* < 0.05). On the other hand, the control group had significantly lower posttest scores in the dimensions of health maintenance efficacy and treatment adherence compared to their pretest scores (*p* < 0.05, Table 4).

**Table 4:** Intragroup comparisons of pretest and posttest scores (*N* = 36).

**Scales**		**Pretest**		**Posttest**		***Z***	***p***	**Effect size (r)**
		**X̄** ±**SD**	**Median (IQR)**	**X̄** ±**SD**	**Median (IQR)**			
**Health-seeking behaviors scale**								
Total HSBS	Control (*n* = 18)	1.57 ± 0.47	1.33 (0.71)	1.00 ± 0.00	1.00 (0.0)	−3.630	**< 0.001**	0.60
	Experiment (*n* = 18)	1.94 ± 0.60	2.04 (1.06)	2.75 ± 0.45	2.83 (0.29)	−3.423	**< 0.001**	0.57
Online health-seeking behaviors	Control (*n* = 18)	1.02 ± 0.08	1.00 (0.0)	1.00 ± 0.00	1.00 (0.0)	−1.000	0.317	0.16
	Experiment (*n* = 18)	1.11 ± 0.32	1.00 (0.0)	1.80 ± 0.72	1.50 (0.71)	−3.450	**< 0.001**	0.57
Professional health-seeking behaviors	Control (*n* = 18)	2.00 ± 0.90	1.67 (0.67)	1.00 ± 0.00	1.00 (0.0)	−3.569	**< 0.001**	0.59
	Experiment (*n* = 18)	2.54 ± 1.10	3.00 (1.75)	4.63 ± 0.34	4.67 (0.42)	−3.691	**< 0.001**	0.61
Traditional health-seeking behaviors	Control (*n* = 18)	2.26 ± 1.14	1.67 (1.50)	1.00 ± 0.00	1.00 (0.0)	−3.330	**< 0.001**	0.55
	Experiment (*n* = 18)	2.98 ± 1.40	3.17 (2.25)	2.76 ± 0.98	3.00 (1.67)	−0.884	0.377	0.14
**Health anxiety inventory**								
Total HAI	Control (*n* = 18)	46.22 ± 9.64	50.00 (6.75)	53.56 ± 1.04	54.0 (0.0)	−3.524	**< 0.001**	0.58
	Experiment (*n* = 18)	45.67 ± 5.68	44.50 (7.50)	7.78 ± 7.68	4.50 (15.0)	−3.726	**< 0.001**	0.62
Excessive concern and anxiety about physical symptoms	Control (*n* = 18)	35.11 ± 9.63	38.50 (8.75)	41.94 ± 0.24	42.0 (0.0)	−3.301	**< 0.001**	0.55
	Experiment (*n* = 18)	34.50 ± 5.61	34.00 (8.25)	7.28 ± 7.20	4.50 (14.25)	−3.725	**< 0.001**	0.62
Negative consequences	Control (*n* = 18)	11.11 ± 1.02	11.00 (1.25)	11.61 ± 0.92	12.0 (0.0)	−1.455	0.146	0.24
	Experiment (*n* = 18)	11.17 ± 1.42	11.50 (1.00)	0.50 ± 0.86	0.0 (1.0)	−3.794	**< 0.001**	0.63
**Chronic illness self-management scale**								
Self-stigma	Control (*n* = 18)	4.57 ± 0.90	5.00 (0.71)	5.00 ± 0.00	5.00 (0.0)	−2.527	**0.012**	0.42
	Experiment (*n* = 18)	4.53 ± 0.64	5.00 (1.0)	1.13 ± 0.28	1.00 (0.04)	−3.752	**< 0.001**	0.62
Coping with stigma	Control (*n* = 18)	1.60 ± 0.79	1.20 (0.90)	1.13 ± 0.31	1.00 (0.0)	−1.960	0.050	0.32
	Experiment (*n* = 18)	1.73 ± 0.49	1.70 (0.65)	4.43 ± 0.41	4.30 (0.85)	−3.732	**< 0.001**	0.62
Health maintenance efficacy	Control (*n* = 18)	1.40 ± 0.75	1.00 (0.50)	1.00 ± 0.00	1.00 (0.0)	−2.232	**0.026**	0.37
	Experiment (*n* = 18)	1.46 ± 0.58	1.00 (1.0)	4.43 ± 0.50	4.50 (0.81)	−3.734	**< 0.001**	0.62
Treatment adherence	Control (*n* = 18)	2.18 ± 0.97	2.00 (0.85)	1.00 ± 0.00	1.00 (0.0)	−3.402	**< 0.001**	0.56
	Experiment (*n* = 18)	2.22 ± 0.62	2.10 (0.65)	4.48 ± 0.69	5.00 (1.40)	−3.730	**< 0.001**	0.62

Z, Wilcoxon Signed-Rank test; X̄, Mean; SD, Standard Deviation.

IQR, Interquartile Range; *p* < 0.05 are in bold.

In the posttest, the experimental group had significantly higher total Health-Seeking Behaviors Scale scores and scores in the Health-Seeking Behaviors scale dimensions of online health-seeking behaviors and professional health-seeking behaviors compared to the pretest, and this difference had a large effect size (*p* < 0.05). Moreover, the experimental group had significantly lower HAI total and dimension scores and Chronic Illness Self-Management Scale self-stigma scores in the posttest than in the pretest, with a large effect size (*p* < 0.05). Additionally, the scores of the experimental group in the coping with stigma, health maintenance efficacy, and treatment adherence dimensions of the Chronic Illness Self-Management Scale were significantly higher in the posttest than in the pretest, and this difference had a large effect size (*p* < 0.05, Table 4).

In the intergroup comparisons of the scale scores of the groups after the intervention, in comparison to the control group, the experimental group was found to have significantly higher posttest Health-Seeking Behaviors Scale total and dimension scores, as well as significantly higher posttest scores in the Chronic Illness Self-Management Scale dimensions of coping with stigma, health maintenance efficacy, and treatment adherence, with a large effect size. Furthermore, with a large effect size, the posttest Health Anxiety Inventory total and dimension scores and the Chronic Illness Self-Management Scale self-stigma dimension scores of the experimental group were significantly lower than those of the control group (*p* < 0.05, Table 5).

**Table 5:** Intergroup comparisons of posttest scores (*N* = 36).

**Scales**	**Control (*****n*** = **18)**		**Experiment (*****n*** = **18)**		***Z***	***p***	**Effect size (r)**
	**X̄** ±**SD**	**Median (IQR)**	**X̄** ±**SD**	**Median (IQR)**			
**Health-seeking behaviors scale**							
Total HSBS	1.00 ± 0.00	1.00 (0.0)	2.75 ± 0.45	2.83 (0.29)	−5.502	**< 0.001**	0.91
Online health-seeking behaviors	1.00 ± 0.00	1.00 (0.0)	1.80 ± 0.72	1.50 (0.71)	−4.792	**< 0.001**	0.79
Professional health-seeking behaviors	1.00 ± 0.00	1.00 (0.0)	4.63 ± 0.34	4.67 (0.42)	−5.537	**< 0.001**	0.92
Traditional health-seeking behaviors	1.00 ± 0.00	1.00 (0.0)	2.76 ± 0.98	3.00 (1.67)	−5.014	**< 0.001**	0.83
**Health anxiety inventory**							
Total HAI	53.56 ± 1.04	54.0 (0.0)	7.78 ± 7.68	4.50 (15.0)	−5.338	**< 0.001**	0.88
Excessive concern and anxiety about physical symptoms	41.94 ± 0.24	42.0 (0.0)	7.28 ± 7.20	4.50 (14.25)	−5.433	**< 0.001**	0.90
Negative consequences	11.61 ± 0.92	12.0 (0.0)	0.50 ± 0.86	0.0 (1.0)	−5.434	**< 0.001**	0.90
**Chronic illness self-management scale**							
Self-stigma	5.00 ± 0.00	5.00 (0.0)	1.13 ± 0.28	1.00 (0.04)	−5.671	**< 0.001**	0.94
Coping with stigma	1.13 ± 0.31	1.00 (0.0)	4.43 ± 0.41	4.30 (0.85)	−5.341	**< 0.001**	0.89
Health maintenance efficacy	1.00 ± 0.00	1.00 (0.0)	4.43 ± 0.50	4.50 (0.81)	−5.502	**< 0.001**	0.91
Treatment adherence	1.00 ± 0.00	1.00 (0.0)	4.48 ± 0.69	5.00 (1.40)	−5.572	**< 0.001**	0.92

Z, Mann-Whitney U test; X̄, Mean; SD, Standard Deviation.

IQR, Interquartile Range; *p* < 0.05 are in bold.

## Discussion

The results of this study showed that the health-seeking behavior education program based on motivational interview techniques increased the health-seeking behaviors of elderly migrant women with chronic diseases, improved their self-management of illness, and reduced their anxiety levels. The results of the study are compared to the results of other studies in the literature with similar populations, interventions, and outcomes, and similarities and differences between these results are discussed in this section.

In the control group in this study, from the pretest to the posttest, there was a significant decrease in the professional and traditional health-seeking behaviors dimension scores of the participants, while a similar trend was also observed in some previous studies. For example, Wong et al. (2020) reported that the health-seeking behaviors of the patients in their cohort did not remain stable over time, and there was a decrease in their access to some healthcare services. According to the authors, this decrease showed that health-seeking behaviors can be affected by environmental or psychological factors in general (Wong et al., 2020). This decrease in the cohort of the aforementioned study may have originated from the challenges experienced by the patients in accessing healthcare services or their lack of knowledge. It should also be noted that this group was affected by the COVID-19 pandemic, which substantially influenced the world for a few years. Similarly, the data in our study were collected in ~1 year after the large-scale Kahramanmaraş earthquakes experienced in Turkey, and this may have led to a gradual increase in the difficulties encountered by the participants in their behaviors of seeking help to protect their health or evaluate the status of their illness. The fact that the large city hospital in Kahramanmaraş was not operational at full capacity at the time of data collection may also explain challenges in help-seeking behaviors. Doherty et al. (2022) stated that the access of elderly individuals to healthcare services varied between heterogeneous groups, this access was particularly limited in those who were not familiar with the system, and this situation could affect health-seeking behaviors. Considering the group with which this study was performed, the effects of cultural and individual differences should not be overlooked. The participants of this study could have reported more negative views in the posttest as they expected more social assistance from the destination country to which they migrated.

The finding in this study that the online health-seeking behaviors of the participants in the control group did not significantly differ from the pretest to the posttest may suggest that internet usage has a more stable effect on access to health-related information. Considering the characteristics of the sample, it would be expected that the online health-seeking behaviors of elderly migrant women would not change without any intervention. In particular, studies on the effects of the internet on health-seeking behaviors showed that internet access usually played an important role in how individuals accessed health-related information (Ma et al., 2023; Pourrazavi et al., 2022). However, whether internet access creates the same effect every time may be associated with how individuals use health-related information obtained from online sources or whether they use this information at all. The participants in the sample of this study may have experienced problems in accessing health recommendations online as a result of their handicaps in terms of technology usage as an older population and their inadequacies in implementing online health recommendations.

The higher HAI total scores and excessive concern and anxiety about physical symptoms dimension scores in the control group in the posttest indicated that the anxiety levels of the participants in this group increased over time. The increase in the anxiety levels in the control group was similar to the result reported by Xiao et al. (2022), who revealed that health anxiety could increase over time in elderly individuals in control groups, and this situation could be associated with their health management strategies. Additionally, Koenig et al. (2012) emphasized that the health anxiety levels of elderly individuals can be affected by environmental stressors and changes in health status. The participants in the control group may have thought that they were not considered healthy enough by the social state and that they did not display behaviors that were sufficiently healthy when they were reached for the pretest, and this may explain the increase in their health anxiety until the posttest.

The increase in the scores of the control group in the Chronic Illness Self-Management Scale dimensions of self-stigma and the decrease in their scores in the health maintenance efficacy and treatment adherence dimensions of the scale in the posttest were similar to the reports in the literature. As stated by Jain (2020), self-stigma is influential on disease management and patient satisfaction, and this may be associated with higher levels of self-stigma in this context. Besides, a decrease in health maintenance efficacy and treatment adherence indicates that the individual experiences problems in adhering to their treatment. Other studies on different factors affecting health maintenance efficacy and treatment adherence presented compatible results (Morton et al., 2015; van Het Bolscher-Niehuis et al., 2016). Morton et al. (2015) argued that the factors that affect treatment adherence in elderly individuals may originate from individual and environmental sources.

The significant rise in the health-seeking behaviors of the participants in the experimental group in this study supported the view that motivational interview techniques can affect the health-seeking behaviors of elderly individuals positively. In a study examining the effects of education programs designed to improve the health-seeking behaviors of elderly individuals, it was observed that such education programs supported the elderly in their processes of seeking health-related information (Gyasi et al., 2019). Likewise, in their study on the health-seeking behaviors of elderly individuals, Sims-Gould et al. (2020) reported that educational interventions had positive effects on seeking health-related information and accessing healthcare services. On the other hand, according to van Het Bolscher-Niehuis et al. (2016), efforts to raise health-seeking behaviors had more limited effects in elderly individuals, and these effects could differ based on personal characteristics such as age, education status, and cultural traits. The more pronounced increases in our study may be attributed to differences in the implementation process of the study and the content of the education program.

In the context of the self-management of illnesses, significant improvements were seen in the scores of the experimental group in the dimensions of self-stigma, coping with stigma, health maintenance efficacy, and treatment adherence. This result demonstrated that motivational interview techniques were effective in improving disease self-management in elderly individuals. Similarly, Ngai et al. (2020) reported that self-management strategies could raise the capacity of elderly individuals to cope with illness. In the study performed by van Het Bolscher-Niehuis et al. (2016), self-management support programs were found to improve health outcomes in the elderly. Nevertheless, it was noted in another study that self-management strategies created limited effects in the elderly, and these effects could vary depending on individual differences and implementation conditions (Alliston et al., 2024). The improvements observed in our study may be explained by differences in the individual characteristics of the participants and the content of the education program.

In this study, there was a significant decrease in the health anxiety levels of the participants in the experimental group. This result showed that motivational interview techniques were effective in reducing the anxiety levels of elderly individuals. Educational programs and psychological support programs were also found effective in reducing health anxiety in elderly individuals with chronic (oncological) diseases (Low et al., 2024). Holvast et al. (2017) also stressed that education programs are important in the management of health anxiety. These similar results suggested that the noticeable decreases in the health anxiety levels of the participants in our study could be associated with the intervention process and individual differences.

### Recommendations

In light of the results of this study, it was determined that the health-seeking behavior education program based on motivational interview techniques was effective in improving health-seeking behaviors and disease self-management in elderly migrant women with chronic diseases. The health information search behaviors and self-management skills of elderly individuals can be increased by expanding such education programs to larger audiences. Hence, it is recommended that healthcare services for elderly migrant women be managed by psychiatric nurses who prioritize and recognize intercultural relations in the context of preventive healthcare services and that collaborations be made with internal medicine nurses to empower the help-seeking behaviors of this population in terms of chronic diseases at the hospital scale. To maintain the positive changes observed in the experimental group, it is recommended that programs to support the health-seeking behaviors and anxiety management of elderly individuals be developed. In particular, additional support can be offered in terms of topics such as health anxiety and coping with stigma. Additionally, to maintain positive outcomes in health-seeking behaviors, coping with health anxiety, and anxiety management, psychiatric nurses are recommended to create support programs in collaboration with internal medicine nurses and ensure the sustainability of such programs. The increase in the online health-seeking behaviors of the experimental group showed that internet usage played an important role in access to health-related information. Therefore, practitioners should aim to increase the access of elderly individuals to the internet and improve their digital literacy. Further studies on the topic in question are needed to understand how effective motivational interview techniques are in different individual and cultural contexts. For instance, the healthcare system of the destination country of elderly migrant women may provide information about these women, and this may allow researchers to explore the discriminations within the system and the boundaries imposed upon health-seeking behaviors by the culture. This could allow programs to be more effectively personalized and adapted. Additional studies can be carried out to investigate the effects of environmental stressors and challenges in access to healthcare services, especially in the context of topics such as health anxiety and treatment adherence. This may allow for a better understanding of obstacles to the health management of elderly individuals.

## Conclusion

The results of this study demonstrated that the health-seeking behavior education program based on motivational interview techniques had significant positive effects among elderly migrant women with chronic diseases. The educational intervention that was carried out in the experimental group increased health-seeking behaviors, improved illness self-management, and lowered the health anxiety levels of the participants. In the experimental group, after the intervention, there were significant improvements in health-seeking behaviors in general and in the context of online and professional health-seeking behaviors. This result showed that health-seeking behavior education programs developed based on motivational interview techniques could affect the health information search behaviors of elderly individuals positively. However, in the control group, no significant change was observed in terms of health-seeking behaviors. Significant improvements were also observed in the experimental group in terms of their self-stigma, coping with stigma, health maintenance efficacy, and treatment adherence scores. Particularly substantial improvements were seen in the context of self-stigma and coping with stigma. These results indicated that the health-seeking behavior education program based on motivational interview techniques was effective in improving the self-management of chronic illness among the participants in the experimental group. In the control group, self-stigma scores increased, and health maintenance efficacy and treatment adherence scores decreased. After the intervention, the health anxiety levels of the participants in the experimental group showed a significant drop. It is seen that the education program implemented in this study had favorable effects on health anxiety. Notwithstanding, in the control group, the health anxiety levels of the participants increased significantly and with a large effect size. As another result, while there was a significant rise in the health-seeking behaviors of the experimental group, the health-seeking behaviors of the control group did not change significantly.

## Data Availability

The raw data supporting the conclusions of this article will be made available by the authors, without undue reservation.
